# Genetic heterogeneity in corpus callosum agenesis

**DOI:** 10.3389/fgene.2022.958570

**Published:** 2022-09-30

**Authors:** Monica-Cristina Pânzaru, Setalia Popa, Ancuta Lupu, Cristina Gavrilovici, Vasile Valeriu Lupu, Eusebiu Vlad Gorduza

**Affiliations:** ^1^ Department of Medical Genetics, Faculty of Medicine, “Grigore T. Popa” University of Medicine and Pharmacy, Iasi, Romania; ^2^ Department of Pediatrics, Faculty of Medicine, “Grigore T. Popa” University of Medicine and Pharmacy, Iasi, Romania

**Keywords:** corpus callosum, agenesis, heterogeneity, chromosomal anomalies, monogenic

## Abstract

The corpus callosum is the largest white matter structure connecting the two cerebral hemispheres. Agenesis of the corpus callosum (ACC), complete or partial, is one of the most common cerebral malformations in humans with a reported incidence ranging between 1.8 per 10,000 livebirths to 230–600 per 10,000 in children and its presence is associated with neurodevelopmental disability. ACC may occur as an isolated anomaly or as a component of a complex disorder, caused by genetic changes, teratogenic exposures or vascular factors. Genetic causes are complex and include complete or partial chromosomal anomalies, autosomal dominant, autosomal recessive or X-linked monogenic disorders, which can be either *de novo* or inherited. The extreme genetic heterogeneity, illustrated by the large number of syndromes associated with ACC, highlight the underlying complexity of corpus callosum development. ACC is associated with a wide spectrum of clinical manifestations ranging from asymptomatic to neonatal death. The most common features are epilepsy, motor impairment and intellectual disability. The understanding of the genetic heterogeneity of ACC may be essential for the diagnosis, developing early intervention strategies, and informed family planning. This review summarizes our current understanding of the genetic heterogeneity in ACC and discusses latest discoveries.

## Introduction

The corpus callosum (CC) is the greatest forebrain commissure in humans, containing approximately two hundred million callosal axons, and its major role is to integrate and transfer information between the two hemispheres. This interhemispheric communication is important for the functions of the cerebral cortex such as integration of sensory, motor, and visuomotor information, abstract reasoning, language processing and management of social and emotional stimuli (Paul et al., 2007; Edwards et al., 2014; Palmer and Mowat 2014; De Leon Reyes et al., 2020). The adult CC is anatomically divided into several regions: rostrum, genu, body, isthmus, and splenium. The rostrum and genu link the frontal and premotor regions of the cerebral cortex, the middle part connects the motor, somatosensory, and parietal regions, while the splenium conjoins the temporal and occipital cortices on both sides (De Leon Reyes et al., 2020).

The aim of this paper is to present the genetic abnormalities involved in the etiology of ACC and their relationship to CC development. We also focused on the suggestive associated features and the benefits of genetic testing for valuable prognostic information and the development of personalized management.

### Literature search strategies and data collection

The data synthesized and presented in this review was obtained by examining the literature (OMIM, PubMed, MEDLINE, Google Scholar databases) and using the following keywords: corpus callosum agenesis, corpus callosal disorders, corpus callosum development, genetic heterogeneity, genetic abnormalities, chromosomal abnormalities, monogenic disorders, chromosomal microarray analysis (CMA), whole exome sequencing (WES), whole genome sequencing (WGS), next generation sequencing (NGS). Only English language papers published within the previous 10 years were considered for this review. A narrative synthesis was generated for the main outcome. The Online Mendelian Inheritance in Man (OMIM) database was used as the reference for gene and mutation symbols.

## Development of the corpus callosum

CC development begins around the 12th week of gestation, and follows well-orchestrated events: neuronal and glial proliferation, neuronal migration and specification, midline patterning, axonal growth and guidance, and post-guidance refinement. The CC is complete and easily recognized by the 14–15th week of gestation whereas the splenium becomes prominent by the 18–19th week of gestation. CC development is a complex process and involves many pathways such as Semaphorin/Plexin/Neuropilin, Slit/Robo, Eph/Ephrin, Netrin/DCC/Unc5, Wnt/Ryk and FGF8/MAPK (De Leon Reyes et al., 2020; Ku and Torii 2020). Some molecules act as nodes of a transcriptional network regulating CC formation. Special AT-rich sequence-binding protein (SATB2), a nuclear matrix DNA-binding protein, involved in transcriptional regulation and chromatin remodeling, plays a central role in development of callosal projections. SATB2 participates in the direct transcriptional repression of *CTIP2* gene (chicken ovalbumin upstream promoter transcription factor-interacting protein 2) (OMIM 606558), and promotes *FEZF2* gene (Fez family zinc finger protein) (OMIM 607414) and *SOX5* gene (OMIM 604975) expression in subcerebral neurons. FEZF2 in turn inhibits high-level *SATB2* gene (OMIM 608148) expression (McKenna et al., 2015; Huang et al., 2020; Ku and Torii 2020). Expressions of *SATB2* and *CTIP2* genes are influenced by histone methylation (e. g. DOT1L), calcium signaling, and other mechanisms (Franz et al., 2019). Molecules from Netrin-1 signaling pathway also interact with *SATB2* gene: *DCC* gene (OMIM 120470) is directly downregulated by SATB2, while *UNC5C* gene (OMIM 603610) is directly upregulated and downregulated by SATB2 and CTIP2 (Srinivasan et al., 2012). The heat shock cognate protein HSC70 is required for the stability of the DCC/TRIO signaling complex and to mediate axon guidance (DeGeer et al., 2015). Netrin-1 can trigger either attraction or repulsion of the axon depending on the receptor it binds. SATB2 also directly upregulates the expression of *EPHA4* gene (OMIM 602118), a member of Eph/ephrin pathways (Srinivasan et al., 2012). Transcription factors FOXG1 promotes *SATB2* gene expression and directly represses *ROBO1* gene (roundabout guidance receptor 1) (OMIM 602430) and *SLIT3* gene (OMIM 603745) expression (Cargnin et al., 2018). Slit molecules bind Robo receptors and act as a repulsive axon guidance cue (Gonda et al., 2020). Amyloid precursor protein (APP) serves as a Slit receptor and mediates axon repulsion (Wang et al., 2017). Semaphorins (SEMA3A and SEMA3C), also have essential roles in corpus callosum development, through signaling mediated by their coreceptors Neuropilin-1 and Plexin-A1 (Hossain et al., 2019).

## Agenesis of the corpus callosum - epidemiology and causes

Many factors could disrupt this complex developmental process and lead to agenesis of the corpus callosum (ACC) also known as corpus callosal disorders. ACC, complete or partial, is one of the most common cerebral anomaly in humans with a reported incidence of 1.8 per 10,000 livebirths, but a significant percentage of prenatally diagnosed cases result in termination of pregnancy or spontaneous abortion (Glass et al., 2008; Weise et al., 2019). The prevalence is higher in children with developmental disabilities: 230–600 per 10,000 (Schell-Apacik et al., 2008; Palmer and Mowat 2014). An increased risk for male gender and preterm birth has been reported (Ballardini et al., 2018). ACC may occur as an isolated anomaly or as a component of a complex disorder, caused by genetic abnormalities, teratogenic exposures or vascular factors. The prevalence of associated anomalies at birth varies considerably among studies, between 39.6% and 86.5% (Glass et al., 2008; Szabo et al., 2011; Stoll et al., 2019). According to Morris et al., 52% of ACC occur as an isolated condition, 25% have associated anomalies (unknown etiology), and 23% are syndromic (with chromosomal, monogenic or teratogenic causes) (Morris et al., 2019). The most common associated anomalies involve the musculoskeletal, the central nervous, the cardiovascular, the urogenital, and the digestive systems (Stoll et al., 2019). The central nervous system anomalies include hydrocephalus, cerebellar hypoplasia, periventricular nodular heterotopia, polymicrogyria, microgyria, and lissencephaly (Glass et al., 2008; Ballardini et al., 2018; Stoll et al., 2019). In cases with hydrocephalus, the differentiation between arrested ventriculomegaly and progressive hydrocephalus is essential for appropriate management. Based on neurological, ophthalmological, and psychometric evaluations, this distinction is very difficult. Radiological markers (frontal-occipital horn ratio, apparent diffusion coefficient, and cerebral blood flow) and intracranial pressure monitoring are very useful diagnostic tools (Leliefeld et al., 2010; Hurni et al., 2018). Genetic causes include complete or partial chromosomal anomalies, autosomal dominant, autosomal recessive or X-linked monogenic disorders, *de novo* or inherited. Several selected syndromes are discussed in more detail concerning their associated anomalies. Multiple other genetic abnormalities are summarized in Tables 1 and 2.

**TABLE 1:** Partial chromosomal anomalies and candidate genes associated with ACC (Dobyns 1996; Schell-Apacik et al., 2008; O'Driscoll et al., 2010; Edwards et al., 2014). The italic values represent genes.

Region	Type	OMIM	Candidate gene
1p36	del	600997 164780 137163 176982	*EPHB2* *SKI* *GABRD* PRKCZ *TMEM52* *CFAP74*
2q14	del	601428 165230 610519	*RNU4ATAC* *GLI2* *CNTNAP5*
2q31-33	del	608148 602188 123810	*SATB2* *EPHA4* *CREB1*
3q24	del	600470	*ZIC1 *
4p16.3	del	134934 602618 616918	*FGFR3* *CTBP1* *PIGG*
5p13-p15	dup	601893	*TRIO*
6p25	dup	601090 603250 612788 615101 612850	*FOXC1* *FOXF2* *FOXQ1* *TUBB2A* *TUBB2B*
9q34.3	del	607001	*EHMT1*
11q 25	dup	116930 608774	*NCAM1* *ANKK1*
13q14	del	604354 603850 611760	*NUFIP1* *PCDH8* *PCDH17*
13q32.3-q33.1	del	603073 617896	*ZIC2* *ZIC5*
13q34	dup	120130 120090 605477 602148 605868	*COL4A1* *COL4A2* *ARHGEF7* *SOX1* *ATP11A*
14q11-q22	del	164874	*FOXG1*
15q24	del	118485 607961 609585 612457 610745 607776	*CYP11A1* *SEMA7A* *CPLX3* *ARID3B* *STRA6* *SIN3A*
16q24.3	del	603464 611192	*CDK10 * *ANKRD11*
17p13.3	del	605066 601545 603527	*YWHAE* *PAFAH1B1* *DPH1*
18q21.2	del	120470	*DCC*
21q22.1-q22.3	del/dup	600855 104760	*DYRK1A* *APP*
22q11.2	del/dup	116790 601754 601180	*COMT* *UFD1L* *RANBP1*
Xp22	del	300552 300118	*MID1* *ARHGAP6*
Xq27.3-q28	dup	300005 300017	*MECP2* *FLNA*

**TABLE 2:** Monogenic syndromes associated with ACC (Edwards et al., 2014; Hofman et al., 2020). The italic values represent genes.

Disease	Gene	Location	Phenotype	OMIM
*Autosomal dominant*				
Mowat Wilson	*ZEB2*	2q22.3	Facial dysmorphism (widely spaced eyes, broad eyebrows with a medial flare, low-hanging columella, prominent or pointed chin, open-mouth expression, uplifted earlobes with a central depression) intellectual disability, epilepsy, Hirschsprung disease, genitourinary anomalies, congenital heart defects, and eye anomalies	235730
Primrose syndrome	*ZBTB20*	3q13.31	Macrocephaly, intellectual disability, enlarged and calcified external ears, facial dysmorphism (high anterior hairline, sparse eyebrows, deeply set eyes, down slanting palpebral fissures, ptosis, high palate, broad jaw), distal muscle wasting and altered glucose metabolism	259050
Sotos syndrome	*NSD1*	5q35.3	Overgrowth, advanced bone age, learning disability, facial dysmorphism (long narrow face, prominent forehead, sparse frontotemporal hair, malar flushing, pointed chin)	117550
Greig cephalo-polysyndactyly	*GLI3*	7p14.1	Macrocephaly, frontal bossing, hypertelorism, pre- or post-axial polydactyly and cutaneous syndactyly	175700
Apert syndrome	*FGFR2*	10q26.13	Multisuture craniosynostosis, midface retrusion, syndactyly of the hands and feet	101200
Baraitser-Winter syndrome	*ACTB* *ACTG1*	7p22.1 17q25.3	Facial dysmorphism (ridged metopic suture, arched eyebrows, hypertelorism, ptosis, broad bulbous nose), ocular coloboma, pachygyria and/or band heterotopias, progressive joint stiffening, intellectual disability, epilepsy	243310 102560
Rubinstein-Taybi	*CREBBP* *EP300*	16p13.3 22q13.2	Intellectual disability, broad thumbs and halluces, facial dysmorphism (highly arched eyebrows, long eyelashes, down slanting palpebral fissures, beaked nose, low hanging columella, high palate, grimacing smile, and talon cusps)	180849 613684
Coffin-Siris	*ARID1A* *ARID1B* *ARID2* *DPF2* *SMARCC2* *SMARCE1* *SMARCA4* *SMARCB1* *SOX4* *SOX11*	1p36.11 6q25.3 12q12 11q13.1 12q13.2 17q21.2 19p13.2 22q11.23 6p22.3 2p25.2	Intellectual disability of varying degree, fifth-digit nail/distal phalanx hypoplasia/aplasia, hypertrichosis, sparse scalp hair, hypotonia, facial dysmorphism (a wide mouth with thick, everted upper and lower lips, broad nasal bridge with broad nasal tip, thick eyebrows, and long eyelashes)	614607 135900 617808 618027 618362 616938 614609 614608 618506 615866

*Autosomal recessive*				
Chudley-McCullough syndrome	*GPSM2*	1p13.3	Early-onset, severe, bilateral sensorineural deafness, brain anomalies (cerebellar dysplasia, hydrocephalus, arachnoid cyst)	604213
Fumarase deficiency	*FH*	1q42.1	Early-onset hypotonia, severe psychomotor retardation, brain abnormalities (cerebral atrophy, ventriculomegaly), seizures, feeding difficulties, increased excretion of fumaric acid in urine	606812
Donnai–Barrow	*LRP2*	2q31.1	Ocular anomalies (high myopia, retinal detachment, progressive vision loss), sensorineural hearing loss, proteinuria, facial dysmorphism (large anterior fontanelle, hypertelorism, enlarged globes, down slanting palpebral fissures, depressed nasal bridge, short nose), intellectual disability, congenital diaphragmatic hernia/omphalocele	222448
Smith-Lemli-Opitz syndrome	*DHCR7*	11q13.4	Prenatal and postnatal growth retardation, microcephaly, intellectual disability, cleft palate, cardiac defects, genital anomalies in males, postaxial polydactyly, syndactyly of the 2nd and 3rd toes, facial dysmorphism (narrow forehead, ptosis, epicanthus, a broad nasal bridge, short nasal root, anteverted nares, micrognathia), elevated serum concentration of 7-dehydrocholesterol	270400
Temtamy syndrome	*C12orf57*	12p13.31	Ocular coloboma, seizures, brain abnormalities (CC and thalamus)	615140
Vici syndrome	*EPG5*	18q12.3-q21.1	Oculocutaneous hypopigmentation, cataracts, progressive cardiomyopathy, variable immunodeficiency, intellectual disability, failure to thrive, myopathy	242840
Fryns syndrome	*PIGN*	18q21.33	Diaphragmatic defect, facial dysmorphism, distal digital hypoplasia, pulmonary hypoplasia, and associated anomalies (polyhydramnios, cloudy corneas/microphthalmia, orofacial clefting, renal dysplasia/renal cortical cysts, and/or malformations involving the brain, cardiovascular system, gastrointestinal system, and/or genitalia)	606097
Microcephaly, short stature, and polymicrogyria (MSSP)	*RTTN*	18q22.2	Microcephaly, intellectual disability, brain malformations (pachygyria, polymicrogyria, midline defects), seizures, microcephaly-related craniofacial dysmorphism (sloping forehead, high and broad nasal bridge), short stature	610436
Joubert	*AHI1* *CPLANE1* *CC2D2A* *CEP290* *CSPP1* *INPP5E* *KIAA0586* *MKS1* *NPHP1* *RPGRIP1L* *TCTN2* *TMEM67* *TMEM216*	6q23.3 5p13.2 4p15.32 12q21.32 8q13.1-q13.2 9q34.3 14q23.1 17q22 2q13 16q12.2 12q24.31 8q22.1 11q13.1	Molar tooth sign, intellectual disability, hypotonia, breathing abnormalities, ataxia, retinal dystrophy, ocular colobomas, renal disease	608629 614615 612285 610188 615636 213300 616490 617121 609583 611560 616654 610688 608091
Meckel	*TMEM216* *TMEM67* *CEP290* *RPGRIP1L* *CC2D2A* *NPHP3* *TCTN2* *B9D1* *B9D2* *TMEM231* *KIF14* *TMEM107* *TXNDC15*	11q12.2 8q22.1 12q21.32 16q12.2 4p15.32 3q22.1 12q24.31 17p11.2 19q13.2 16q23.1 1q32.1 17p13.1 5q31.1	Brain malformation (occipital encephalocele, hydrocephalus, anencephaly, holoprosencephaly, Dandy-Walker malformation), cystic renal disease, postaxial polydactyly, hepatic abnormalities, liver fibrosis	613277 609884 610142 610937 612013 608002 613846 614144 611951 614949 611279 616183 617778
Hydrolethalus	*HYLS1* *KIF7*	11q24.2 15q26.12	Central nervous system malformation (hydrocephalus, keyhole-shaped foramen magnum, agenesis of midline structures) preaxial polydactyly in the hands and feet, hallux duplex, heart defects, respiratory tract abnormalities	610693 611254

*X-linked*				
Craniofrontonasal dysplasia	*EFNB1*	Xq13.1	Craniofacial asymmetry, coronal craniosynostosis, frontal bossing, hypertelorism, depressed nasal bridge, bifid nasal tip, frizzy and curly hair, longitudinally ridged fingernails	304110
L1syndrome	*L1CAM*	Xq28	Hydrocephalus of varying degrees of severity, intellectual disability, spastic paraplegia, adducted thumbs	307000 303350 304100
Opitz-Kaveggia syndrome (FG1 syndrome)	*MED12*	Xq13.1	Intellectual disability, hypotonia, constipation, feeding problems, characteristic behavior (affable, eager to please), broad thumbs and halluces, facial dysmorphism (relative macrocephaly, prominent forehead with frontal hair upsweep, hypertelorism, down slanting palpebral fissures, and open mouth)	305450
Lujan-Fryns syndrome	*MED12*	Xq13.1	Intellectual disability, hypotonia, tall stature, marfanoid habitus, hyper-extensible fingers and toes, hypernasal speech, facial dysmorphism (long narrow face, high and narrow nasal bridge, short philtrum, highly arched palate)	309520

*Unknown causative gene/mode of inheritance*				
Toriello-Carrey syndrome	*unknown*		Intellectual disability, Pierre Robin anomaly, facial dysmorphism (telecanthus, short palpebral fissures, small nose with anteverted nares, abnormally shaped ears), heart defects, distal limb defects, urogenital anomalies, postnatal growth delay	217980

## Chromosomal anomalies

Chromosomal abnormalities have been identified in 4.81–17.8% of ACC; especially when associated congenital anomalies are present. The reported complete aneuploidies include: trisomy 18, 13, 8 in mosaic, 21 and X monosomy (Glass et al., 2008; O'Driscoll et al., 2010; Palmer and Mowat 2014; D'Antonio et al., 2016). Callosal abnormalities are also associated with partial chromosomal anomalies, the most common: 8p inverted duplication/deletion syndrome, 1q43q44, 6q27 and 17q21.31 microdeletions are characterized bellow. However, many copy number variants have been reported in cases with ACC (Table 1).

### 1q43q44 microdeletion syndrome

1q43q44 microdeletion syndrome (OMIM 621337) is a disorder characterized by moderate to severe intellectual disability with limited or no expressive speech, microcephaly, agenesis/hypogenesis of the CC, seizures, suggestive facial dysmorphism (round face, prominent forehead, hypertelorism, epicanthal folds, flat nasal bridge, and malformed and low-set ears), and hand and foot anomalies. Balliff et al. proposed a 75 Kb critical region for ACC that contains *ZBTB18* gene (OMIM 608433), which encodes a transcriptional repressor of key genes involved in neuronal development (Ballif et al., 2012). The phenotypic heterogeneity (no ACC) in cases with critical region deletion has been explained by incomplete penetrance or variable expressivity. Depienne et al. considered that ACC is also influenced by the alteration of neighboring genes especially *HNRNPU* (OMIM 617391) (Ballif et al., 2012; de Munnik et al., 2014; Cohen et al., 2017; Depienne et al., 2017).

### 8p inverted duplication/deletion syndrome [invdupdel (8p)]

Interstitial inverted duplication 8p associated with 8pter deletion is a complex and rare chromosomal rearrangement. Clinical features include mild to severe intellectual disability, hypoplasia/agenesis of the CC, characteristic facial dysmorphism (prominent forehead, temporal baldness, anteverted nostrils, eversion of the lower lip, large mouth and ears), skeletal anomalies (scoliosis/kyphosis), hypotonia, and congenital heart defects. The invdupdel (8p) consists of a deletion of the telomeric region (8p23-pter) followed by an intermediate intact fragment, and a proximal inverted duplication of variable size. Rearrangements are mediated by two highly repetitive regions: the olfactory receptor gene clusters or defensin repeat (ORDRs); the polymorphic 8p23 inversion between these clusters increases the susceptibility of 8p to rearrangements. The 8p23.2-pter region contains the genes *ARHGEF10* (OMIM 608236) and *CSMD1* (OMIM 608397), associated to central nervous system development (de Die-Smulders et al., 1995; Giglio et al., 2001; Garcia-Santiago et al., 2015; Redaelli et al., 2022).

### 6q27 microdeletion

6q27 deletion is associated with a variable phenotype including intellectual disability, seizure, hypotonia, brain malformations (ACC, periventricular nodular heterotopia (PNH), polymicrogyria, hydrocephalus, and cerebellar malformations), short neck and craniofacial dysmorphism (microcephaly, broad nose with prominent nasal root and bulbous nasal tip, large and low-set ears, and downturned mouth). Other commonly reported findings are: joint laxity, heart defects and retinal anomalies. The size of deletion varies between 0.4 Mb and 10.8 Mb and does not seem to correlate with phenotype severity, suggesting the existence of a minimal critical region. Hanna et al. proposed a 325 kb critical region for brain malformation and intellectual disability, comprising the *DLL1* (OMIM 606582), *PSMB1* (OMIM 602017), *TBP* (OMIM 600075), and *PDCD2* (OMIM 600866) genes (Hanna et al., 2019)*.* The key role of *DLL1* is supported by cases with brain anomalies, intellectual disability and pathogenic variants in *DLL1* gene*. DLL1* gene is Notch ligand with an important role in development of the central nervous system (regulates neuronal differentiation), somites and lymphocytes (Striano et al., 2006; Peddibhotla et al., 2015; Fischer-Zirnsak et al., 2019; Hanna et al., 2019; Lesieur-Sebellin et al., 2022).

### 17q21.31 microdeletion

17q21.31 microdeletion known as Koolen-de Vries syndrome (KDVS) (OMIM 610443) is characterized by intellectual disability, hypotonia, suggestive facial dysmorphism (upslanting palpebral fissures, blepharophimosis, ptosis, epicanthus, a pear-shaped nose with bulbous nasal tip, and large/protruding ears), friendly behavior, brain anomalies (ventriculomegaly, ACC, hydrocephalus, Arnold-Chiari type I malformation), seizures, joint hypermobility, heart defects, and genitourinary anomalies. KDVS is caused by a 17q21.31 microdeletion or by a pathogenic variant in the *KANSL1* gene (OMIM 612452). Genotype-phenotype correlation studies did not report significant clinical differences between microdeletions and pathogenic variant patients. The recurrent 17q21.31 microdeletion breakpoints map to flanking extensive low copy repeats suggesting that deletion is mediated by non-allelic homologous recombination. *KANSL1* encodes the KAT8 regulatory NSL complex Subunit 1, a member of a histone acetyltransferase complex, which regulates global transcription (Koolen et al., 2008; Zollino et al., 2012; Koolen et al., 2016).

## Monogenic disorders

Monogenic causes have been identified in 8–35% of ACC (Bedeschi et al., 2006; Schell-Apacik et al., 2008; Palmer and Mowat 2014; Ballardini et al., 2018; Morris et al., 2019). Advances in genetic sequencing technologies have enabled the discovery of pathogenic variants in a large number of genes: from those involved in development of callosal projections to genes encoding components of microtubules or metabolic enzymes (Table 2) (Schell-Apacik et al., 2008; Palmer and Mowat 2014; Ballardini et al., 2018; Morris et al., 2019).

### Acrocallosal syndrome (ACS)

ACS (OMIM 200990) is a ciliopathy characterized by ACC, distal limb anomalies (postaxial polydactyly of the hands, and preaxial polydactyly of the feet), intellectual disability and facial dysmorphism (prominent forehead, hypertelorism, short nose with broad nasal bridge). In ACS the most frequent mutations were found in *KIF7* gene (15q26.1) (OMIM 611254) while mutations of *GLI3* gene (7p14.1) (OMIM 165240) are rare (Elson et al., 2002; Putoux et al., 2011). Both genes are involved in the ciliary sonic hedgehog (SHH) pathway. Depletion of proteins related to SHH pathway is associated with ciliogenesis defects. *KIF7* gene encodes a member of kinesin motor proteins family, that localize from the base to the tip of the cilium and plays important roles in the regulation of microtubule acetylation and stabilization, and SHH signaling (Niceta et al., 2020). *GLI3* gene encodes a zinc finger transcription factor, that interacts with microtubule associated MID1 protein and KIF7 and has a dual function: as a transcriptional activator and a repressor of SHH pathway (Laclef et al., 2015). Biallelic mutations in *KIF7* gene have been reported in Hydrolethalus syndrome and rare cases of Joubert syndrome, with overlapping features, suggesting a continuous phenotypic spectrum. A severe phenotype is associated with *KIF 7* gene truncating mutations, while missense *KIF7* gene variants have been reported in milder cases (Putoux et al., 2011). Asadollahi et al. reported mutation in *KIF7, SHH*, and *CPLANE1* genes in patients with suggestive facial dysmorphism for ACS and ciliopathy features (Asadollahi et al., 2018). Monoallelic mutations in *GLI3* gene have been reported in Greig cephalopolysyndactyly and Pallister Hall syndromes. A group of ciliopathies associate intellectual disability and brain malformations, including ACC, highlighting the critical role for primary cilia in brain development (Valente et al., 2014; Arghir et al., 2021; Hasenpusch-Theil and Theil 2021).

### Tubulinopathies

Tubulinopathies comprise a wide spectrum of brain malformations (lissencephaly, cerebellar hypoplasia, ACC, pachygyria, dysgyria), variable degree of motor and intellectual disabilities and epilepsy, caused by pathogenic variants in tubulin genes (α, β and γ isotypes) (Romero et al., 2018). The alpha and beta families of tubulin genes encode proteins which form heterodimers as fundamental components of microtubules. Tubulins interact with other proteins (e.g. β-III-tubulin connects with DCC in a netrin-1 dependent manner) and play key roles in neuronogenesis, neuronal migration, cortical organization–pial basement membrane integrity, and also in axon guidance (Qu et al., 2013; Boyer and Gupton 2018). All mutations reported in *TUBA1A* (12q13.12, OMIM 602529), *TUBB2B* (6p25.2, OMIM 612850)*, TUBB3* (16q24.3, 602,661) and *TUBB* (6p21.33, 1919130) genes are heterozygote missense variants; this could suggest a dominant-negative effect. *TUBA1A* gene missense variants are the most frequent; some hot spots have been reported: Arg264 (c.790C>T, predominantly results in central pachygyria, whereas c.791G>A causes microlissencephaly with complete agenesis of the corpus callosum), Arg402 and Arg422 (Romaniello et al., 2015; Hebebrand et al., 2019).

### *FOXG1* syndrome

*FOXG1* syndrome (OMIM 613454), is a rare neurodevelopmental disorder, with a wide spectrum of features ranging from severe intellectual disability with poor or absent speech development, postnatal microcephaly, corpus callosum anomalies (agenesis/hypogenesis), delayed myelination, seizures, and dyskinesia, to deficient social interactions, disrupted circadian rhythm, and autistic traits. It has been previously classified as a congenital variant of Rett syndrome, due to clinical features and evolution, including a normal perinatal period followed by a phase of developmental regression at the age of 3–6 months. *FOXG1* gene (OMIM 164874), located at 14q12, encodes the forkhead box protein G1, a conserved transcriptional repressor, that play a critical role in forebrain development, from the timing of neurogenesis, to the patterning of the cerebral cortex and callosal projections. *FOXG1* gene truncating mutations (frameshift or nonsense) in the N-terminal domain and 14q12 microdeletions associate a severe phenotype, while *FOXG1* gene missense mutations in the forkhead domain lead to milder phenotype (Kortum et al., 2011; Leombroni et al., 2018; Mitter et al., 2018; Hou et al., 2020; Akol et al., 2022).

### ACC–Neuropathy syndrome (Andermann syndrome)

Hereditary motor and sensory neuropathy with ACC (OMIM 218000) is a channelopathy characterized by severe progressive sensorimotor neuropathy with resulting hypotonia, areflexia, and amyotrophy, and by dysgenesis of the corpus callosum. Common features include intellectual and developmental delays, psychiatric manifestations (paranoid elements, depression, hallucinations), progressive scoliosis and facial dysmorphism (palpebral ptosis, hypertelorism, hypoplastic maxilla, high-arched palate). It is inherited in an autosomal recessive manner, and the high incidence in the Saguenay and Lac-Saint-Jean regions of Quebec, Canada, due to the c.2436+1delG mutation in *SLC12A6* gene (OMIM 604878) suggest a founder effect. *SLC12A6* gene, located at 15q14, encodes a solute carrier family 12 member 6 (KCC3), a potassium chloride cotransporter, expressed in the peripheral and central nervous system. Impaired KCC3 function alters maintenance of homeostasis of the membrane threshold, leading to neuronal degeneration. A case with a gain of function mutation and peripheral neuropathy but no corpus callosum abnormalities has been reported (Gauvreau et al., 1993; Kahle et al., 2016; Flores et al., 2019).

### Aicardi syndrome

Aicardi syndrome (OMIM 304050) is characterized by a triad of features: agenesis or hypogenesis of corpus callosum, chorioretinal lacunae, and infantile spasms. Other common findings include: periventricular and subcortical heterotopia, intracranial cysts, polymicrogyria, retinal and optic nerve colobomas, microphthalmia, costovertebral anomalies, gastrointestinal disorders (reflux, constipation, feeding difficulties) and suggestive facial dysmorphism (prominent premaxilla, upturned nasal tip, decreased angle of the nasal bridge, and sparse lateral eyebrows). It affects usually females, but the disease was identified in few cases with Klinefelter syndrome (47, XXY) or 46, XY males with profound microcephaly. The inheritance of Aicardi syndrome is considered X-linked dominant with early embryonic lethality in the majority of hemizygous males. Skewed X chromosome inactivation and translocations involving Xp22 have been reported in several cases but a candidate gene has not been identified. Nemos et al. excluded mutations in the *CDKL5* gene (Xp22.13) (OMIM 300203) (Eble et al., 2009; Nemos et al., 2009; Wong and Sutton 2018).

### Corpus callosum agenesis - Abnormal genitalia syndrome (Proud syndrome)

Corpus callosum agenesis - abnormal genitalia syndrome (OMIM 300004) is characterized by ACC, neurological manifestations (intellectual disability, developmental delay, epilepsy, spasticity), and urogenital anomalies (hypospadias, cryptorchidism, renal dysplasia). It is part of a broad phenotypic spectrum of disorders caused by mutation in the *ARX* gene (OMIM 300382), ranging from lissencephaly to ACC with abnormal genitalia to developmental and epileptic encephalopathy without brain malformations to syndromic and nonsyndromic X-linked intellectual disability. *ARX* gene, located at Xp22.13, encodes a transcription factor which belongs to the class of homeobox proteins. *ARX* participate to fundamental processes of brain development: patterning, neuronal proliferation and migration, cell maturation and differentiation, axonal outgrowth and connectivity. ARX contains several domains, including the homeobox domain, a PRD-like domain, a N-terminal octapeptide domain, a central acidic domain, a C-terminal aristaless domain, as well as three nuclear localization sequences and four polyalanine tracts. Premature termination mutations and missense variants in the homeodomain or nuclear localization sequences are associated with severe phenotype involving brain malformations. Missense mutations outside of the homeobox domain or expansions in the polyalanine tracts lead to milder phenotype without brain malformation. Usually, males are severely affected, and heterozygous females may be unaffected or have a milder phenotype (Kato et al., 2004; Friocourt et al., 2006; Olivetti and Noebels 2012; Ghadami and Eshaghkhani 2019; Leung et al., 2022).

### Pyruvate dehydrogenase complex (PDC) deficiency

PDC deficiency (OMIM 312170, 614111, 245348, 245349, 246904, 608782) is an inborn error of mitochondrial energy metabolism, with a wide phenotypic spectrum ranging from fatal neonatal lactic acidosis to neurological manifestations (hypotonia, seizures, ataxia and developmental delay) with structural brain abnormalities. ACC is found in over one-third of patients with *PDHA1* gene mutations (Pavlu-Pereira et al., 2020; Savvidou et al., 2022). The disorder can result from mutations in multiple genes that encode components of the PDC: *PDHA1* (Xp22.1) (OMIM 300502), *PDHB* (3p14.3) (OMIM 179060), *DLAT* (11q23.1) (OMIM 608770), *PDHX* (11p13) (OMIM 608769), *DLD* (7q31.1) (OMIM 238331) and *PDP1* (8q22.1) (OMIM 605993). The most common are mutations in the *PDHA1* gene, which encodes E1 alpha subunit, and has eleven exons. Frameshift *PDHA1* gene mutations occur preferentially in exons 10 and 11, associate a very low enzyme activity and severe phenotype. Missense *PDHA1* gene mutations are located usually in exons one to nine and are more prevalent in males. Hemizygous males are generally symptomatic and more severely affected (with significant lethality in infancy) whereas heterozygous females present variable clinical manifestations and greater survival due to random patterns of X-inactivation (Patel et al., 2012; Horga et al., 2019; Pavlu-Pereira et al., 2020).

### Pyridoxine-dependent epilepsy (PDE)

PDE (OMIM 266100) is characterized by recurrent seizures, usually with early onset, resistant to standard anticonvulsants but with clinical and electroencephalogram (EEG) improvement following pyridoxine supplementation. Intellectual disability is common (75%–80%) and does not correlate with seizure control or age at treatment initiation (Gospe et al., 2021; Coughlin et al., 2019). Callosal abnormalities are the most frequent neuroimaging findings and are not explained by treatment lag (Toldo et al., 2018; Tseng et al., 2022). PDE is caused by mutation in *ALDH7A1* gene (OMIM 107323), located on 5q23.2, which encodes alpha-aminoadipic semialdehyde (α-AASA) dehydrogenase, a key enzyme in lysine metabolism. The enzyme deficiency, inherited in an autosomal recessive manner, leads to accumulation of α-AASA, pipecolic acid, and Δ-1-piperideine-6 carboxylate, responsible for sequestration of pyridoxal-5-phospate. Missense mutations are the most common and are more frequent in exons 5 and 6 (within the linker domain), 10 (within the NAD binding domain), 15 (within the catalytic domain). The most prevalent mutation is c.1279G>C (pGlu427Gln) and homozygotes patients present variable phenotype with neonatal or late-onset. Splice site (e.g. c.834G>A), frameshift and nonsense mutations have been also reported (Coughlin et al., 2019). The deep intronic mutation c.696–502G>C results in the usage of two cryptic splice sites and introduction of a pseudoexon between exons 7 and 8 (Milh et al., 2012). Coughlin et al. hypothesized that some mutations affect not only the enzyme activity but also the stability or the ability to reach its targeted environment (Coughlin et al., 2019).

### Congenital mirror movements (CMM)

CMM (OMIM 157600) is characterized by early onset, involuntary movements of one side of the body that mirror intentional movements on the opposite side. Physiologic mild mirror movements are occasionally found in young children, but their persistence beyond the age of 7 years is pathologic. CMM usually persist throughout life and involve predominantly the upper limbs, more severe distal (Meneret et al., 2014). Monoallelic truncating (frameshift and nonsense) mutations of *DCC* gene (OMIM 120470) are associated with CMM. Missense mutations c.2378T>G (p.Val793Gly) and c.2414G>A (p.Gly805Glu), located within the NTN1 binding interface are also associated with CMM. Monoallelic *DCC* gene mutations are associated with variable expressivity and incomplete penetrance. The *DCC* gene, located at 18q21.2, encodes a functional receptor for netrin (NTN1), a transmembrane glycoprotein belonging to the immunoglobulin (Ig) superfamily of cell adhesion molecules. DCC plays a key role in the transduction of NTN1-induced attractive and repulsive signaling in the coordinated outgrowth and guidance of commissural axons that cross the anatomical midline of the body. Corticospinal axons and callosal axons use slightly different signaling pathways, and a *DCC* mutation may differentially affect commissural versus subcerebral axon trajectories, explaining the variable phenotypes: mirror movements, ACC, or both (Srour et al., 2010; Marsh et al., 2018). CMM is also caused by monoallelic mutation in *NTN1* gene (17p13.1) (OMIM 601614)*, RAD51* gene (15q15.1) (OMIM 179617) and biallelic mutations in *DNAL4* gene (22q13.1) (OMIM 610565), but no callosal anomalies have yet been reported (Ahmed et al., 2014; Meneret et al., 2014).

### Horizontal gaze palsy with progressive scoliosis (Split brain syndrome) (OMIM 617542)

Biallelic, *DCC* gene mutations lead to a complex syndrome associated with a broad disorganization of white-matter tracts throughout the CNS (Jamuar et al., 2017). The features include absence of all commissures (including the CC, anterior and posterior), brainstem defects (including hypoplasia of the pons and midbrain), horizontal gaze palsy, impaired intellectual development and progressive scoliosis. Biallelic mutations are associated with complete penetrance (Jamuar et al., 2017; Marsh et al., 2018). Biallelic mutation in *ROBO3* gene (11q24.2) (OMIM 608630), which encodes a coreceptor of NTN1, leads to horizontal gaze palsy with progressive scoliosis and brainstem defects **(**hypoplasia of the pons and cerebellar peduncles, butterfly configuration of the medulla, and pontine cleft in the midline); no CC anomalies have been reported. ROBO3 is a member of the roundabout family of receptors, inhibits ROBO1/2, interacts with DCC and potentiate commissural axonal outgrowth and attraction (Jen et al., 2004; Pinero-Pinto et al., 2020).

### Septo-optic dysplasia (SOD)

SOD (OMIM 182230) is characterized by a clinical triad of optic nerve hypoplasia, dysfunction of the hypothalamic-pituitary axis, and midline brain abnormalities, including agenesis of the septum pellucidum and CC. For a SOD spectrum diagnosis, a combination of any two of the three features is required. Only 30%–47% of patients present the complete classical triad. Additional findings include developmental delay, seizures, cerebral palsy, autism, sleep disorders, thermoregulatory disturbances, hearing loss, anosmia, and heart defects. Associated brain abnormalities (hydrocephalus, polymicrogyria, grey matter heterotopias) have also been reported (sometimes referred to as SOD-plus syndrome) (Webb and Dattani 2010; Ganau et al., 2019; Ward et al., 2021). SOD is usually sporadic but familial cases with intrafamilial variability have been reported. Genetic abnormalities have been identified in <1% of the patients. Homozygous or heterozygous mutations in *HESX1* (3p14.3) (OMIM 601802) have been described in patients with variable phenotypes ranging from isolated growth hormone deficiency to SOD. *HESX1* encodes a paired-like homeobox transcription factor, which acts as a transcriptional repressor and is essential for normal forebrain and pituitary development. Heterozygous *HESX1* mutations are associated with a milder phenotype and incomplete penetrance (Kelberman and Dattani 2008; Webb and Dattani 2010; Fang et al., 2016). Mutations in other genes involved in the regulation of the Wnt/β-catenin signaling pathway have been reported in SOD cases: *SOX2* (3q26.33) (OMIM 184429), *SOX3* (Xq27.1) (OMIM 313430), *OTX2* (14q22.3) (OMIM 600037), *TCF7L1* (2p11.2) (OMIM 604652) or *TAX1BP3* (17p13.2) (OMIM 616484). *SOX2* mutations are associated with severe eye abnormalities, and additional features (genital anomalies, esophageal atresia, intellectual disability, seizures) (Blackburn et al., 2018). *SOX3* duplications/point mutations have been reported in cases with hypopituitarism and/or intellectual disability (Arya et al., 2019; Li et al., 2022). *OTX2* pathogenic variants are associated with hypopituitarism and eye abnormalities and incomplete penetrance (Gregory et al., 2021). Mutations in genes involved in hypogonadotropic hypogonadism with or without anosmia (OMIM 612702) - *FGFR1* (8p11.23) (OMIM136350) and *FGF8* (10q24.32) (OMIM 600483) have also been described in SOD patients suggesting a genetic overlap (Raivio et al., 2012). The etiology of SOD remains unclear in the majority of patients; vascular abnormalities, teratogens, and young maternal age may also be involved (Ganau et al., 2019).

## Isolated ACC

The genetic causes of isolated ACC appear more elusive but some pathogenic variants in genes associated with syndromic ACC have been reported. *DCC* gene missense mutations, located within the NTN1 binding sites (in fourth, fifth and sixth fibronectin type III-like domains), are associated with isolated ACC, in females. Marsh et al. observed a significant dose-dependent increase in *DCC* gene expression in testosterone-treated neural stem cells derived from human embryonic stem cells and suggested that isolated ACC may occur when *DCC* gene expression is below a critical level during CC development. Jouan et al. identified compound heterozygous missense variants in the *CDK5RAP2* gene (OMIM 608201) in 3 sibs with isolated ACC. The *CDK5RAP2* gene, located at 9q33.2, encodes cyclin-dependent kinase 5 regulatory subunit 2 (CDK5RAP2) a pericentriolar structural protein that has a major role in the microtubule-organizing function of the centrosome through interaction with the *γ*-tubulin ring complex. Its expression is particularly high within the brain, especially within the thalamus and corpus callosum (Jouan et al., 2016). These findings suggested that alteration of *CDK5RAP2* activity may dysregulate neurogenic cell divisions. Biallelic mutations in *CDK5RAP2* are associated with autosomal recessive primary microcephaly and isolated ACC. Jouan et al. hypothesized that mutations with residual function may lead to isolated ACC, while severe loss-of-function variants (nonsense, splice site, truncating missense) may result in severe microcephaly with reduced brain size and concomitantly with partial or complete ACC (Jouan et al., 2016). The factors determining the phenotypic variability are complex and include the type and location of mutation and possible genetic modifiers (Jouan et al., 2016; Marsh et al., 2018).

**FIGURE 1:**
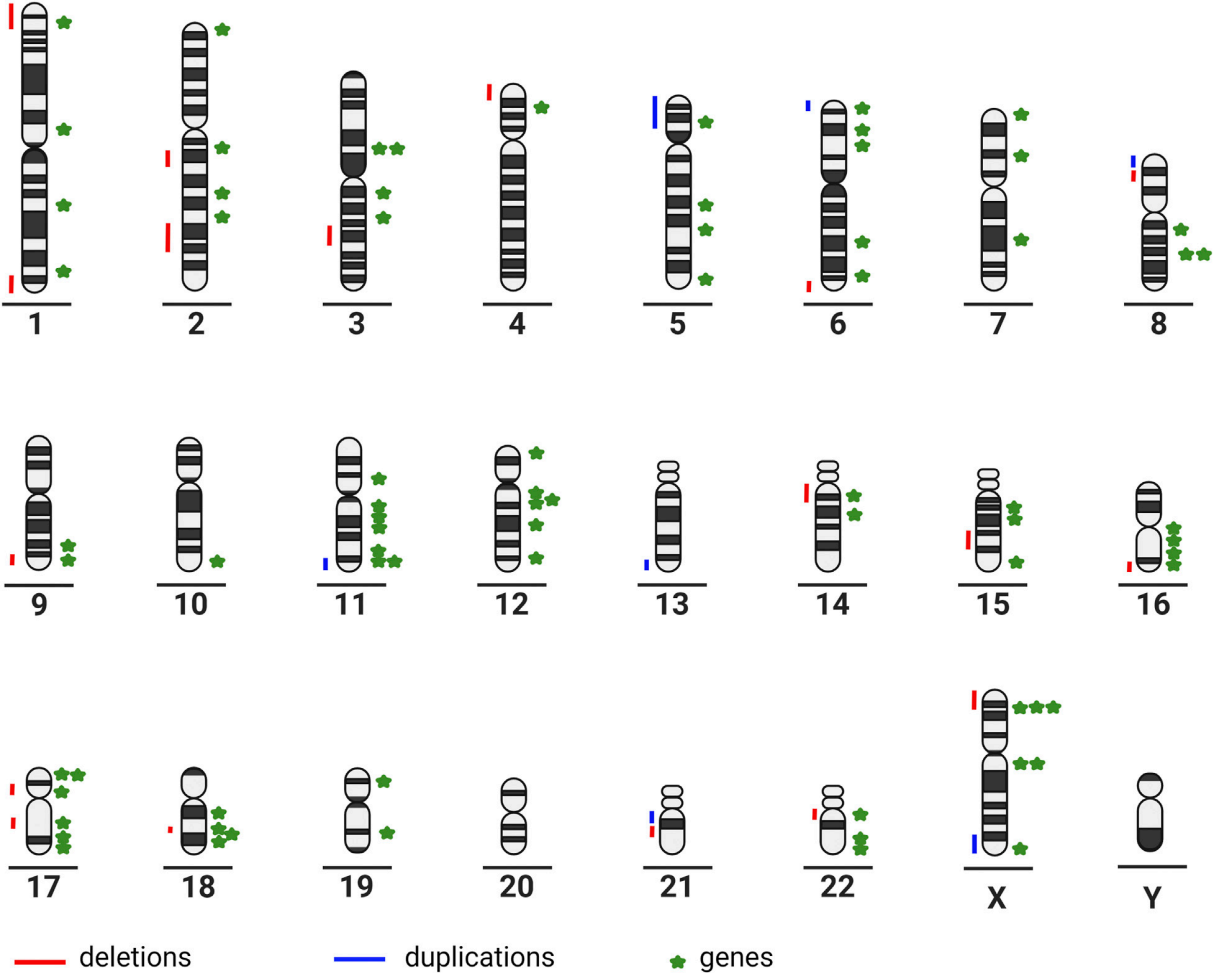
Chromosome ideograms illustrating genetic heterogeneity: partial chromosomal anomalies are represented on the left-hand side of the ideogram; genes associated with ACC are represented on the right-hand side of the ideogram (created in BioRender–publication license May-30–2022); (Chromosome 1*: ARID1A, GPSM2, KIF14, FH.* Chromosome 2: *SOX11, NPHP1, ZEB2, LRP2.* Chromosome 3*: HESX1, PDHB, ZBTB20, NPHP3.* Chromosome 4*: CC2D2A.* Chromosome 5*: CPLANE1, ALDH7A1, TXNDC15, NSD1.* Chromosome 6*: TUBB2B, SOX4, TUBB, AHI1, ARID1B.* Chromosome 7*: ACTB, GLI3, DLD.* Chromosome 8: *CSPP1, TMEM67, PDP1.* Chromosome 9*: CDK5RAP2, INPP5E.* Chromosome 10*: FGFR2.* Chromosome 11*: PDHX, DPF2, TMEM216, DHCR7, DLAT, HYLS1, ROB O 3.* Chromosome 12*: C12orf57, ARID2, TUBA1A, SMARCC2, CEP290, TCTN2.* Chromosome 14*: FOXG1, KIAA0586.* Chromosome 15*: SLC12A6, RAD51, KIF7.* Chromosome 16*: CREBBP, RPGRIP1L, TMEM231, TUBB3.* Chromosome 17*: TMEM107, NTN1, B9D1, SMARCE1, MKS1, ACTG1.* Chromosome 18*: EPG5, DCC, PIGN, RTTN.* Chromosome 19*: SMARCA4, B9D2.* Chromosome 22*: SMARCB1, DNAL4, EP300.* Chromosome X*: CDKL5, ARX, PDHA1, EFNB1, MED12, L1CAM*)*.*

The large number of partial chromosomal anomalies and monogenic syndromes associated with ACC illustrate the high genetic heterogeneity (Figure 1).

## Prognosis

In isolated ACC, neurodevelopmental outcomes range from normal development in about 64.8–75% of the individuals to different levels of intellectual disability, including severe (in 12% of cases) (D'Antonio et al., 2016; Bernardes da Cunha et al., 2021). Sleep disturbances are also common and include: greater sleep onset delay, decreased sleep duration, sleep anxiety, night wakings, parasomnias, sleep-disordered breathing, daytime sleepiness, increased slow-wave sleep, more distressful dreams, and narcolepsy (Ingram and Churchill 2017; Yates et al., 2018). Regular follow-up is necessary because some cases with initial “normal development” presented subtle speech, attention, and reasoning difficulties with increasing age. In a significant percentage of cases (approximately 40%) with ACC, social and behavioral problems have been reported: emotional immaturity, impaired social competence, deficits in planning, and poor communication of emotions (Wegiel et al., 2017). While autistic elements have been reported in cases with ACC, MRI in patients with autism revealed thinning of corpus callosum but further studies are necessary to elucidate the connection between corpus callosum anomalies and autism (Frazier and Hardan 2009; Paul et al., 2014; Hofman et al., 2020). Other adverse outcomes related to ACC include epilepsy, visual problems and psychiatric disorders (schizophrenia) (Popoola et al., 2019). The rate of severe disability in cases with associated anomalies is higher than that in patients with isolated ACC. The spectrum of neurodevelopmental outcome depends on several factors, such as the extension of the ACC (complete or partial), the presence and severity of associated defects, as well as on the magnitude of residual interhemispheric transfer through other commissures (Guadarrama-Ortiz et al., 2020). A checklist of suggested diagnostic evaluations in a postnatal patient with ACC is summarized in Table 3.

**TABLE 3:** Suggested diagnostic evaluations in a postnatal patient with ACC.

No:	Evaluations
1	MRI
2	Thorough physical examination
3	Neurological evaluation
4	Ophthalmic examination
5	Neuropsychological evaluation
6	Ultrasound of the urogenital tract
7	Cardiac evaluation
8	Gastrointestinal evaluation
9	Hearing evaluation
10	Genetic testing
11	Specific investigations: metabolic (if a metabolic disorder is suspected), congenital infections

The variable neurodevelopmental outcomes of ACC make prenatal counseling a challenge. Fetal MRI plays a critical role in detection of associated anomalies, a major prognosis determinant (Bernardes da Cunha et al., 2021). Some studies reported discrepancy between prenatal diagnosis of isolated ACC and postnatal or postmortem diagnosis of additional anomalies (5% in complete ACC and 15% in partial ACC). These discrepancies may be related to the technology, method of diagnosis, or to the timing of investigations because some anomalies may be identified only in the third trimester of pregnancy (Paladini et al., 2013; Griffiths et al., 2017; Leombroni et al., 2018).

Prenatal genetic diagnosis is essential in these cases. Chromosomal abnormalities (except mosaicism if abnormal cells are not present in the examined specimen) and an increasing number of monogenic disorders can be diagnosed prenatally. Conventional prenatal karyotype identifies complete or large partial aneuploidies in 4.8%–7.5% of ACC (Leombroni et al., 2018). Prenatal chromosomal microarray analysis (CMA) in cases with normal karyotype reveals pathological copy number variations in 5.7–6.9% of ACC (Greenbaum et al., 2021). However, the diagnostic rate of fetal karyotype and CMA does not exceed 15–20%, because monogenic causes have the highest frequency. In this context, many studies highlighted the utility of WES in determining the genetic etiology of CCA including in prenatal cases. The detection rate is higher in cases with associated anomalies. WES can also detect many copy number variations. Gene variant interpretation could be challenging due to incomplete prenatal phenotype (Reches et al., 2018; Heide et al., 2020; Lei et al., 2021; She et al., 2021).

## Conclusion

Intensive research over the last decade has highlighted the genetic heterogeneity of ACC, which reflects the complexity of CC development. Although an impressive number of chromosomal abnormalities and gene mutations have been reported in ACC–associated syndromes, there are still conditions of unclear etiology. Incomplete penetrance and variable expressivity, unexplained by the type of mutation, suggest the existence of genetic modifiers. Detection of associated anomalies and genetic causes is essential in prenatal detected cases, given the variable outcome, oscillating between the normal cognitive level and severe psychomotor delay. WES alone or simultaneous, WES and CMA may be considered a first-tier test in the prenatal diagnosis of ACC given the frequency of monogenic and chromosomal anomalies and the time constraints. The elucidation of all molecular mechanisms of ACC and the genotype-phenotype correlations will aid in understanding of CC role and in development of personalized management.
